# Community-Based Management: Under What Conditions Do Sámi Pastoralists Manage Pastures Sustainably?

**DOI:** 10.1371/journal.pone.0051187

**Published:** 2012-12-11

**Authors:** Vera H. Hausner, Per Fauchald, Johnny-Leo Jernsletten

**Affiliations:** 1 Department of Arctic and Marine Biology, University of Tromsø, Tromsø, Norway; 2 Department of Arctic Ecology, Norwegian Institute for Nature Research, FRAM Centre, Tromsø, Norway; 3 Centre for Sámi Studies, Teorifagbygget, Tromsø, Norway; University of Massachusetts, United States of America

## Abstract

Community-based management (CBM) has been implemented in socio-ecological systems (SES) worldwide. CBM has also been the prevailing policy in Sámi pastoral SES in Norway, but the outcomes tend to vary extensively among resource groups (“siidas”). We asked why do some siidas self-organize to manage common pool resources sustainably and others do not? To answer this question we used a mixed methods approach. First, in the statistical analyses we analyzed the relationship between sustainability indicators and structural variables. We found that small winter pastures that are shared by few siidas were managed more sustainably than larger pastures. Seasonal siida stability, i.e., a low turnover of pastoralists working together throughout the year, and equality among herders, also contributed to more sustainable outcomes. Second, interviews were conducted in the five largest pastures to explain the relationships between the structural variables and sustainability. The pastoralists expressed a high level of agreement with respect to sustainable policies, but reported a low level of trust and cooperation among the siidas. The pastoralists requested siida tenures or clear rules and sanctioning mechanisms by an impartial authority rather than flexible organization or more autonomy for the siidas. The lack of nestedness in self-organization for managing pastures on larger scales, combined with the past economic policies, could explain why CBM is less sustainable on the largest winter pastures. We conclude that the scale mis-match between self-organization and the formal governance is a key condition for sustainability.

## Introduction

Community-based management (CBM) has been promoted as a sustainable alternative for governing common pool resources, such as pastures, forests, water and fisheries [1]–[3]. The endorsement of CBM is based on extensive empirical research showing that local users have successfully devised their own systems of rules and sanctions for the sustainable harvesting of resources [3], [4]. Over the past two decades, policy reforms related to CBM have also been advocated by international development agencies, the European Community, and the Convention on Biological Diversity. Many of these reforms bear references to the subsidiary principle, which argues that local users have a better understanding of the socio-ecological systems (SES) they manage and greater incentives to manage their own resources sustainably; management authority, therefore, should be transferred to the lowest appropriate level [5], [6]. As the experience with the devolution reforms and CBM in terms of delivering sustainable outcomes has been equivocal, the focus has shifted towards analyzing conditions likely to influence the SES [1], [6], [7]. Ostrom suggested to analyze the factors affecting sustainable SES before crafting sustainable solutions [6]. For CBM, this means analyzing causes that affect the local users’ ability to work towards sustainable outcomes collectively.

The conventional approach has been to consider the size of the resource system, the group size and the socioeconomic heterogeneity as the key conditions for the sustainable management of SES [1], [8]. In particular, small and homogenous groups of resource users have been expected to act collectively toward common goals, but the empirical evidence supporting this claim is inconclusive [8], [9]. To better understand how such variables affect the likelihood of cooperation, including their interaction with other structural factors, there is a need to link these variables to explanatory mechanisms [10]. The literature related to the concept of social capital suggests that, in small communities, dense social networks and frequent interactions, should build mutual trust among resource users over time [11]. Repeated interactions build reputations for being trustworthy, and higher levels of trust make it more likely that individuals build reciprocal relationships and collaborate repeatedly. Other studies claim that trust is insignificant as a cause of cooperation, and that leadership or institutional mechanisms that require, forbid or permit and sanction specific actions result in cooperation despite low levels of trust [12], [13]. Such institutions could also be informal and based on social norms, where a bad reputation and the fear of losing social bonds may encourage individuals to cooperate [10], [14].

To self-organize for sustainable resource management, resource users must have a minimum degree of autonomy and secure access to the resource [4], [8]. However, autonomy and the community’s ability to make and enforce its own rules could be undermined by cross-scale linkages beyond the control of the local users [1], [7], [14]. CBM strategies that were once effective can be challenged by outside forces, resulting in overharvesting and the breakdown of cooperation in local communities [2], [3], [14]. Furthermore, as the spatial scale of the resource or group size increases, there arises a need for a stronger endogenous organization to enable cooperation [4]. Whereas larger cross-scale cooperation can emerge endogenously among users through their self-organization into multiple nested levels [4], there is often a need for higher levels of governance for the formal recognition of community rules, sanctioning, or conflict resolution among the users [1], [5], [7].

In this study, we focus on the question posed by Elinor Ostrom: “Why do some resource users self-organize to manage common pool resources sustainably and others do not?” [3], [8]. This question has mainly been addressed by small-N studies or game theoretical approaches, which do not permit the testing of the relative importance of different factors in explaining sustainable outcomes. Poteete et al., therefore, call for more engagement in broadly comparative and large-N research to evaluate the generality of different hypotheses [8]. Here, we adopt a mixed methods design that combines statistical modeling and semi-structured interviews to link the relative importance of structural variables with explanatory mechanisms [8], [15], [16]. Such interdisciplinary approaches to sustainability have also been argued as essential to overcome some of the challenges in the Sámi pastoralist SES [17], [18].

**Figure 1:**
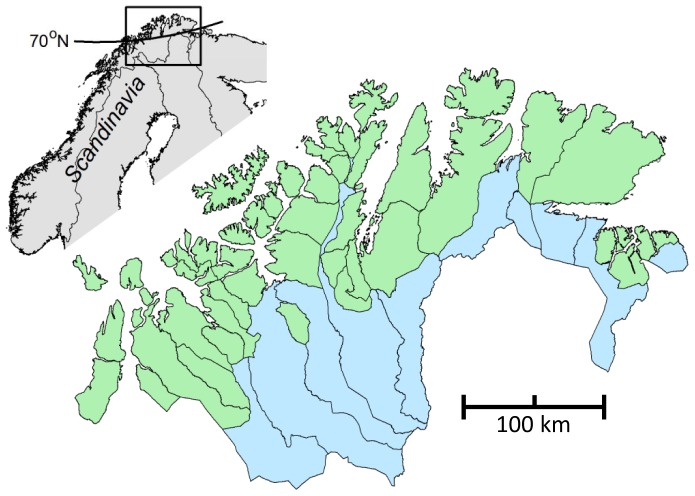
Study area. Green areas indicate the delineation of summer pasture areas and blue indicate winter pasture areas in 2003/04. Most of the summer pastures (39 of the 44) are managed by one siida. In winter many of the summer siidas are split into smaller winter siidas that share winter pastures.

We build on a rather unique dataset which allows for comparative quantitative research on the management of reindeer pastures by Sámi pastoralists in Finnmark, Norway. The pastoralists self-organize in groups (siidas) in which the reindeer are herded together [19]–[21]. Usually the siida is based on kinship and is both a social and working association. The siida institution varies in form and flexibility: some pastoralists herd together all year, whereas other siidas group and regroup during the course of the year depending on seasonal pastures. The sustainability of the Sámi pastoralist SES depends on the management of both summer and winter pastures (Text S1, Figure 1). On the summer pastures one siida usually has the exclusive rights to well-defined pastures, and therefore the pastoralists usually let the reindeer roam freely to exploit the heterogeneous resources. The siida organization on winter pastures is more variable. Specifically, five large winter pastures are managed by many siidas, whereas five other pastures are managed by only a few siidas. During winter, access to grazing resources varies according to snow condition, but in order to avoid inter-mixing with neighboring herds, continuous herding with snowmobiles is often necessary in the areas with many siidas. Sustainable outcomes in such SES are likely to depend on the match or mismatch between the scale of self-organization and the scale of formal governance. It has been argued, on the other hand, that the sharing of large pastures by several groups, i.e., with fluid and overlapping boundaries between herders, adds a level of flexibility to respond to environmental variability and, therefore, is more fit for such SES [22]–[25]. Flexible herding and livestock mobility have also been emphasized as important for coping with climate variability in the Arctic pastoralist SES [17], [26]–[28]. The outcomes of such flexible organizations depend on the degree of trust and the pastoralists’ ability to negotiate and cooperate on a larger scale.

To explore how the organization of herders might affect sustainability, we first examined the relationships between indicators of sustainable outcomes and structural variables including: i) the area of the pastures, ii) the inequality of herders with respect to herd size, and iii) seasonal siida stability, i.e., the turnover of pastoralists working together throughout a year. Secondly, the explanatory mechanisms behind these relationships were explored by field studies on the largest winter pastures. The semi-structured interviews focused on the pastoralists’ understanding of the resource situation, trust and institutional arrangements. On the basis of our results, we discuss how the CBM strategies in Norway (see Text S1) that emphasize i) the devolution of rulemaking authority to the lowest level of organization, ii) well-defined boundaries and iii) the creation of clear rules and sanction mechanisms fit the Sámi pastoralist SES.

## Materials and Methods

### Quantitative Analysis of Structural Variables

Data on individual herding units were mostly derived from the ecological statistics compiled annually by the Norwegian reindeer husbandry administration [29]. We analyzed data from the West and East Finnmark reindeer herding areas (Figure 1) for a ten-year period (1998 to 2007). Because we use the spatial variation to analyze the SES, we removed the noise associated with temporal changes by averaging the data for each herding unit over the ten year period (for analyses of time series; see [30]). We removed the smallest units that kept less than 20 reindeer. The final dataset comprised 292 herding units.

In Finnmark, as much as 39 of the 44 summer pastures are each managed by one siida. The remaining five summer pastures are each shared by two or three siidas. We used the delineation of siida pastures to indicate summer pasture size. In contrast, the five largest winter pastures encompass 11–21 siidas, whereas the five smallest winter pastures are managed by one to six siidas each (Figure 1). We related indicators of sustainable outcomes to structural variables in mixed linear regression models. We used three different indicators of sustainability as response variables: i) proportion of calf slaughter ii) calf body mass, and iii) livelihood income. The proportion of calves slaughtered reflects sustainable herding practices [30]–[32]. Slaughtering calves, instead of waiting until the animals are one year of age or older, reduces the herd size during the winter and therefore the grazing pressure and the losses due to starvation on the winter pastures. Calf body mass is the carcass mass measured at the slaughterhouses. In our study area there is a negative impact of high animal density on the quality of the summer pastures [33] and, as a consequence, a strong negative relationship between calf body mass and reindeer density [30], [31]. High calf body mass reflects high level of growth during the summer, animals in good body condition and reduced vulnerability to harsh winter conditions [30], [31], [34], [35]. The livelihood economy indicator is a measure of the economic benefits derived from the pastures, which is the sum of income from the sale of slaughtered animals and governmental disbursements through slaughter subsidies (provided by the Reindeer Husbandry Administration) and compensation for losses to predators (provided by the Directorate for Nature Management).

We used the pasture area, inequality among herders and seasonal siida stability as structural predictor variables in the analyses. We expected the pasture size to be an important determinant of sustainability. However, due to difference in the match between the pasture size and the siida organization, we expected the effect of pasture size to differ between summer and winter pastures. Inequality has been hypothesized to influence incentives for collaboration and, therefore, sustainable outcomes [8]. Previous studies of Sámi pastoral communities have documented inequalities associated with the herd size, both in terms of the access to resources and power [19], [21], [36]. Based on the number of reindeer possessed by each herding unit, we used the Gini coefficient as a measure of equality [37]. The Gini coefficient ranges from 0, indicating perfect equality, to 1, indicating maximal inequality, and we expected low Gini coefficients to be associated with more sustainable outcomes. The seasonal stability index reflected the turnover of pastoralists working together throughout the year. For each herding unit, the index was calculated by dividing the number of herding units cooperating year-round by the total number of herding units cooperating on either the summer or winter pastures. This index range from 0, indicating that no units herd together year-round, to 1, indicating that all pastoralists herd together year-round (i.e. stable siida partnership, Text S1). Because individual herd size is an important determinant of individual success in Sámi pastoral communities [38], we also included the individual herd size as a covariate in the analyses.

The data were analyzed by linear mixed-effect models (*lme*) using the *nlme* library in *R* [39], [40]. For each of the three response variables we constructed an initial model with a given set of structural predictor variables (fixed factors). The response variables were measured on the herding unit level, giving 292 numbers of observations in each model. The initial models included six fixed factors: herd size, siida stability, winter Gini, summer Gini, winter size and summer size. Herd size and siida stability were measured on the herding unit level. The Gini coefficients and pasture sizes were measured on the winter pasture level (winter Gini and winter size) and on the summer pasture level (summer Gini and summer size). The 44 summer pastures were nested within the 10 winter pastures, i.e.; herders from different summer pastures shared the same winter pasture, while no herders from different winter pastures shared the same summer pasture. Summer pasture nested within winter pasture was accordingly included as a random factor in the models.

The degrees of freedom of the fixed factors was determined by the level at which the factor was measured. Therefore, due to different degrees of freedom, the relative importance of the fixed factors cannot be assessed by their associated *t* and *P*-values. To compare the relative importance of the different coefficients, we standardized each variable to a mean equal to zero and a standard deviation equal to one. The effect of a coefficient b_1_ from a predictor X1 with standard deviation s_1_, on a response variable Y with standard deviation s_Y_, should be interpreted as follow: An increase of s_1_ in X1 results, on average, in an increase in b_1_*s_Y_ in Y.

We used the Akaike’s Information Criterion (AIC) to investigate how model simplification affected the relationships revealed by the initial models [41]. From a pool of candidate models containing all the possible combinations of the six fixed factors, we selected the model with the lowest AIC. The random structure was held constant for all candidate models.

### Interview Inquiry of Explanatory Mechanisms

The quantitative analyses showed that unsustainable outcomes were more common in the five largest winter pastures indicating that collective action problems might be more pronounced in these areas. Accordingly, we selected the five largest winter pasture areas for the interview inquiry (for design and methods see http://www.ecologyandsociety.org/vol16/iss3/art4/appendix3.html, Accessed 2012 Nov 12, [30]). We asked: Q1. Is there agreement among pastoralists about sustainability policies? Q2. Is there trust among pastoralists? Q3. What is the role of institutional arrangements? In most cases, we coded the interviews on three ordinal levels. “Not Available” (NA) was used in all cases where the variables could not be coded due to unclear answers or no response. The number of respondents (N), therefore, varied for each variable.

#### Q1. Is there agreement among pastoralists about sustainability policies?

One hypothesis for the lack of community solutions is the occurrence of disagreements about the resource situation among the pastoralists. Reduction of the number of reindeer and pastoralists has been the major policy goal in the last 20 years [30], [42]. Agreement on the overall goal that reindeer numbers should be adjusted to pasture capacity to increase the condition of the animals, therefore, was a major question (Q1.1). The policies also aim to change herding practices to increase the production per hectare, particularly by reducing the herd sizes (Q.1.2) and stimulating calf slaughter (Q1.3) [30], [42]. Lastly, meat production for market and the maximization of economic income has not traditionally been the aim of Sámi pastoralists, and a lack of adaptation could, therefore, be due to their inclination for non-market values (Q.1.4) [19], [21], [36]. We coded policy agreement on three levels: disagree, partly agree and agree.

#### Q2. Is there trust among pastoralists?

Trusting other pastoralists to reciprocate is assumed to be essential for cooperation [8], particularly in those systems in which the flexible use of pastures demands continuous negotiations between the pastoralists [22]–[24]. We coded trust on three levels for both the summer and winter pastures (Q2.1 and 2.2). Trust was coded as “no” for pastoralists keeping their herds away from neighboring pastoralists or claims of negative reciprocity, such as suspicious losses of reindeer, changes of earmarks or keeping reindeer too long on others pastures. Trust was coded as weak when the pastoralists responded that they communicate but are unsure about the others’ intentions or disagree about their decisions. On the summer pastures, we additionally coded trust as weak for claims of other pastoralists not contributing sufficiently to labor or financial costs. Full trust of other pastoralists was coded as strong in situations where the pastoralists reported good communication and positive reciprocity, such as informing other pastoralists of lost reindeer and returning the reindeer or letting them remain on their pastures for a period if needed.

#### Q3. What is the role of institutional arrangements?

We hypothesized that outcomes on the five largest pastures could be explained by a mismatch between the scale of self-organization (the siidas) and the scale of formal governance (i.e. the need for well-defined boundaries) or, a lack of sufficient autonomy for the pastoralists to make their own decisions. Voluntary programs for establishing customary tenures have been initiated by the administration, but formalizing customary rights through independent courts have also been discussed (see Text S1, [28], [43]). We therefore asked about the need for customary tenures on the winter pastures and whether such a division should be based on negotiations or formalized by a court (Q3.B). The need for clearly defined rights on the winter pastures depends on the ability of the users themselves to negotiate and resolve conflicts related to pasture use. Local arenas that allow for rapid conflict resolution may increase the levels of trust and the degree of cooperation among the users [4]. We therefore asked about the existing conflict resolution mechanisms (No, Negotiation, or Formalized) (Q3.A). The high degree of participation in rulemaking has been shown to increase the likelihood of sustainable management [44]. However, to solve the problems of collective action among users, the higher levels of governance must themselves be trustworthy [11]. We coded the pastoralists’ trust to the multilevel co-management system as no, weak or strong levels of trust (Q3.C). Lastly, we asked if more autonomy is needed at the level of the summer siidas (Q3.D).

### Ethics Statement

Our standards for ethics were approved by the Norwegian Social Science Data Services and included a letter of information about the study before the start of the interview to secure voluntary participation and a perusal of the final transcript of the interview, if requested. We informed about the survey, the purpose and how data would be stored and used. Due to abuse of written statements in the past, Sámi elders are reluctant to provide signatures of any kind. Thus we obtained verbal consent from the participants. This procedure was approved by the Norwegian Social Science Data Services, which check the ethics and is the only institution that issue official licenses to all social science studies in Norway.

## Results

### Statistical Analyses

**Figure 2:**
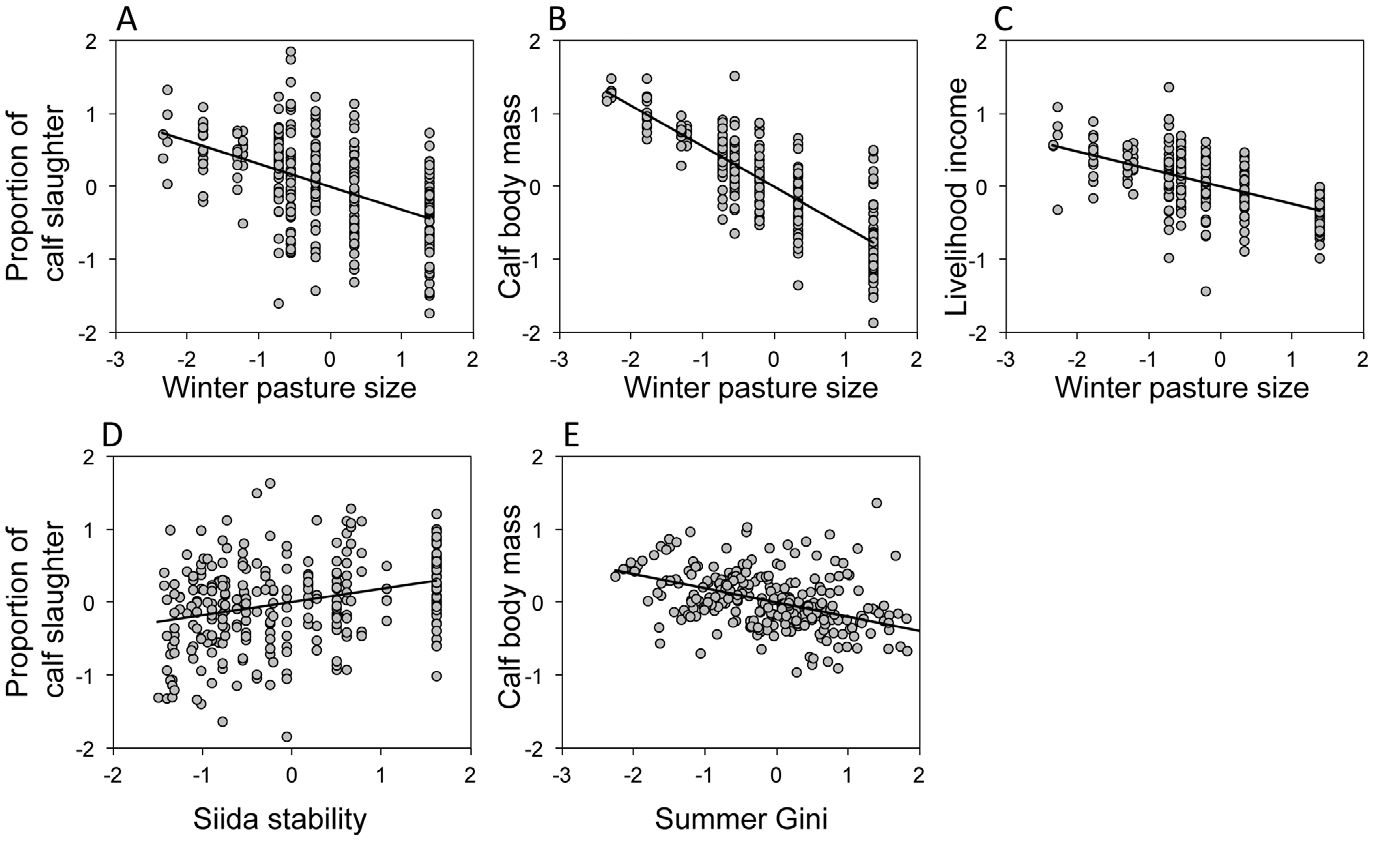
Relationships between structural variables and sustainability indicators selected by AIC. Partial residual plots between (A) winter pasture area and proportion of calf slaughter, (B) winter pasture area and slaughter weights of calves, (C) winter pasture area and livelihood income, (D) seasonal siida stability and proportion of calf slaughter, and (E) inequality in herd size on the summer pasture (Gini coefficient) and slaughter weight of calves. Each point is the average value of a herding unit from 1998 to 2007. The partial residuals were computed from the initial mixed effect model, relating the sustainability indicators to six structural predictor variables (see Table 1). To allow comparisons of effect sizes, all variables were standardized to mean equal to zero and standard deviation equal to one.

The size of the winter pasture had, relative to the other structural variables, a strong negative effect on all the sustainable outcome variables (Table 1; Figure 2A, 2B, 2C). The effect was however, only marginally significant for the proportion of calf slaughter (P = 0.048) and not significant for calf body mass (P = 0.066). However, model simplification based on AIC retained the effect of winter pasture size in all the analyses. The effects were significant (P<0.05) in all the selected models, suggesting that winter pasture size was an important factor explaining sustainable outcomes. In contrast, the effects of summer pasture size were relatively weak and variable, and this factor was consistently removed by the model selection procedure (Table 1). There was a significant positive relationship between the seasonal siida stability and the proportion of calf slaughter (Table 1; Figure 2D). This result shows that herders, on average, slaughtered a higher proportion of calves when they were organized into the same siida throughout the year. With respect to the calf body mass, the Gini coefficient on summer pastures had a negative effect (Table 1; Figure 2E), indicating that equality in herd size among herders on the summer pastures was associated with higher slaughter weights of calves. Finally, we found a positive relationship between herd size and income and between herd size and the proportion of calf slaughter (Table 1).

**Table 1:** Mixed-effect models of sustainability outcomes.

A) Proportion of calf slaughter					
Fixed effects	Estimate	S.E.	DF	t-value	P
Herd size	0.134	0.042	246	3.22	0.002
Stability	0.181	0.063	246	2.86	0.005
Winter size	−0.314	0.131	7	−2.39	0.048
−/Summer size	0.233	0.157	32	1.49	0.147
−/Winter Gini	−0.220	0.108	7	−2.04	0.081
−/Summer Gini	−0.032	0.091	32	−0.35	0.728
**Random effects**				**StDev**	**nObs**
Among winter pastures				0.10	10
Among summer pastures (within winter pastures)				0.60	44
Residuals				0.59	292
**B) Calf body mass**					
**Fixed effects**	**Estimate**	**S.E.**	**DF**	**t-value**	**P**
−/Herd size	−0.078	0.030	246	−2.55	0.011
−/Stability	−0.106	0.049	246	−2.18	0.030
Winter size	−0.558	0.256	7	−2.17	0.066
−/Summer size	−0.117	0.224	32	−0.52	0.605
−/Winter Gini	0.158	0.200	7	0.79	0.456
Summer Gini	−0.370	0.094	32	−3.93	0.000
**Random effects**				**StDev**	**nObs**
Among winter pastures				0.07	10
Among summer pastures (within winter pastures)				0.61	44
Residuals				0.58	292
**C) Livelihood income**					
**Fixed effects**	**Estimate**	**S.E.**	**DF**	**t-value**	**P**
Herd size	0.811	0.023	246	35.60	0.000
−/Stability	0.064	0.035	246	1.80	0.073
Winter size	−0.242	0.071	7	−3.40	0.012
−/Summer size	0.071	0.089	32	0.80	0.432
−/Winter Gini	−0.075	0.060	7	−1.25	0.252
−/Summer Gini	0.026	0.052	32	0.49	0.626
**Random effects**				**StDev**	**nObs**
Among winter pastures				0.00	10
Among summer pastures (within winter pastures)				0.35	44
Residuals				0.32	292

(A) proportion of calf slaughter (B) slaughter weights of calves, and (C) livelihood income. The response variables were modeled with respect to (fixed factors): herd size, seasonal siida stability, pasture size (summer and winter size) and the inequality in herd size (summer and winter Gini). Summer pasture nested within winter pasture were modeled as random factors. To allow comparisons of effect sizes, all variables were standardized to mean equal to zero and standard deviation equal to one prior to the analyses.

−/Fixed effects removed by model simplification based on AIC.

### Interview Inquiry

**Figure 3:**
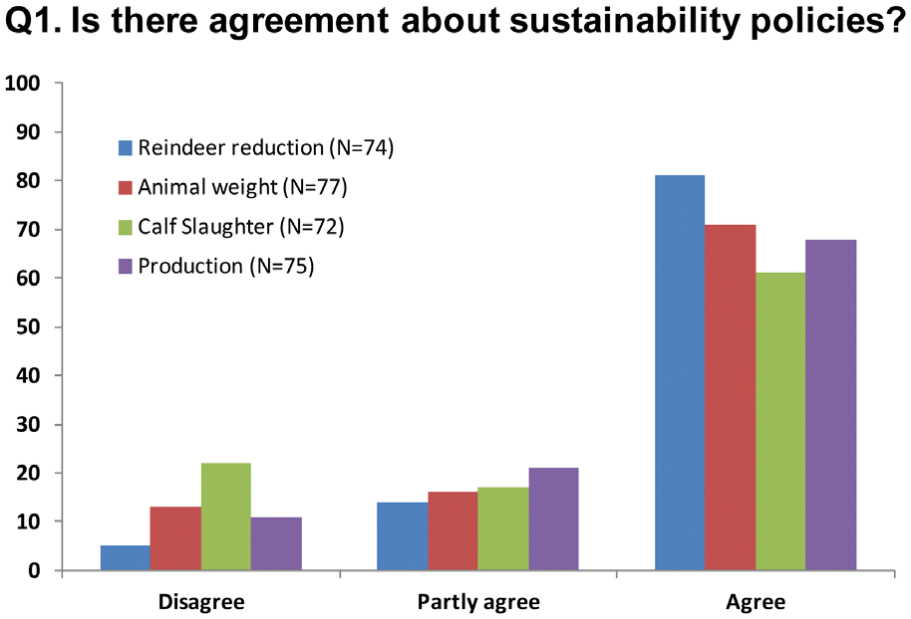
Agreement about sustainability policies. Percentages of the pastoralists who disagreed, partly agreed and agreed with sustainability policies.

The interviews suggested that the variables used as outcome variables in the statistical analyses were in agreement with the pastoralists’ own views on sustainability (Q1, Figure 3). There was strong support for an adjustment of reindeer numbers to the pasture capacity in which higher weights are preferable to keeping large herds. However, most of the pastoralists indicated the need for others to reduce their herds; i.e. either small owners who have alternative income or large owners who keep very large herds. The majority preferred to slaughter the surplus calves before moving on to the winter pastures to reduce the risk of losses and to improve production outcomes. The focus on production was also strongly supported by the pastoralists, who reported meat production as their primary goal. Most of the respondents argued that the costs of reindeer pastoralism are high, thus to cover the expenses for snowmobiles and housing, they needed to think in terms of production and income. This does not mean, however, that non-market factors were unimportant. The pastoralists claimed that the prevailing cause for keeping large herds was to gain informal influence (71%) and to access the best winter pastures (66%).

**Figure 4:**
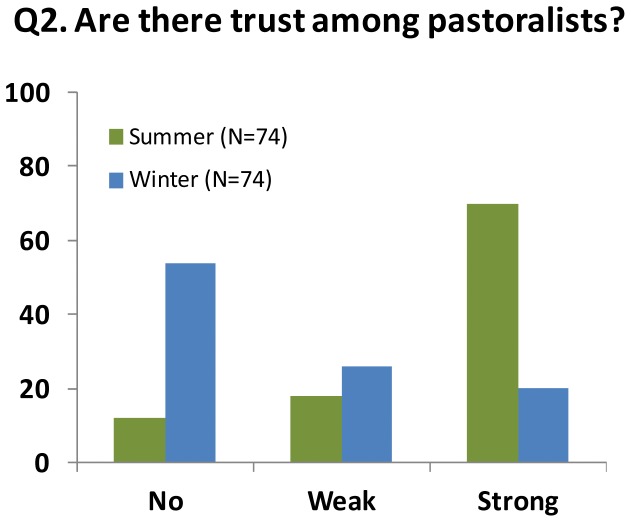
Trust among pastoralists. Percentages of the pastoralists who expressed no, weak and strong degrees of trust for other pastoralists sharing winter and summer pastures.

There was a clear difference in the levels of trust for the summer and winter pastures (Q2, Figure 4). Most summer pastures were managed by one siida group in which a high degree of trust was based on strong family ties and a long history of collaboration. A lack of trust was more evident on the winter pastures, as 52% of the respondents are suspicious to their neighbors, and only 19% have a strong degree of trust for their neighboring herdsmen. According to the older pastoralists, there have always been flexible boundaries on the winter pastures, potentially leading to tension between neighbors, but they formerly had a core tenure associated with their winter siida groups that was generally respected by the other pastoralists. The flexible boundaries were necessary for the adaptation to shifting pasture conditions, such as icing or deep snow, that limit the access to lichen. Many of those who experienced conflicts explained that the 1978 Reindeer Husbandry Act named the winter pastures as “common pastures” which, according to the respondents, had not been the case previously. The system of customary tenures was consequently not respected by some of the pastoralists who expanded their herds into these areas (see also Text S1). Keeping large herds bestows influence and access to the best winter pastures, providing incentives for some pastoralists to increase their herds.

**Figure 5:**
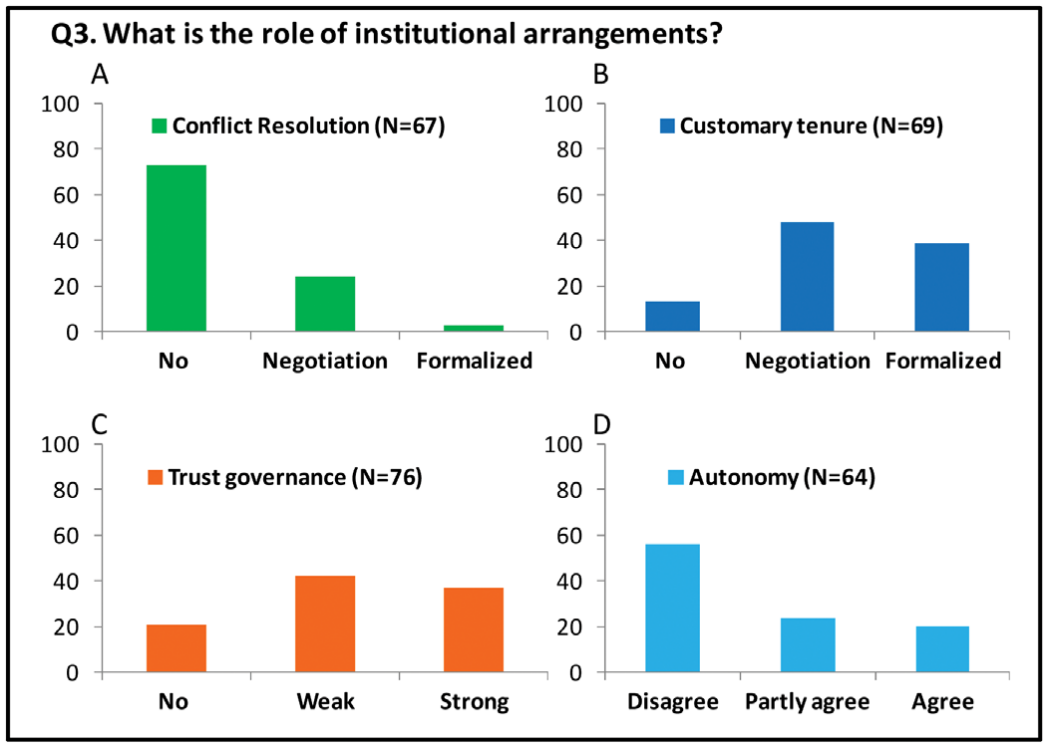
Institutional arrangements. The pastoralists’ perceptions of institutional arrangements. A) Percentages of the pastoralists who reported no, negotiated and formalized conflict resolution mechanisms. B) Percentages of the pastoralists who responded negatively to the idea of dividing the pastures into customary tenures or responded positively to the idea of establishing customary tenures, either by voluntary agreements or court formalization. C) Percentages of the pastoralists who expressed no, weak and strong degrees of trust in the current governance regime. D) Percentages of the pastoralists who disagreed, partly agreed and agreed that more autonomy is needed to achieve sustainability.

The lack of conflict resolution mechanisms for the winter pastures was striking (Q3.A, Figure 5). The small herders explained that the only options are to move away from the large herds to avoid losing too many reindeer. In conflict areas, the pastoralists spend most of their time herding reindeer on snowmobiles to avoid the mixing of herds. Traditionally, conflicts have been solved through negotiation, and some respondents referred to informal leaders, “siida isit,” who used to confer with neighbors on the winter pastures. There is a special police unit, the reindeer police, who mitigate and resolve conflicts among the pastoralists related to such activities as gathering, separation, marking and slaughtering. However, the pastoralists considered it inappropriate to report anyone to the police, and some pastoralists explained that such a report could bring retribution and “losses” of reindeer. The respondents also argued that neither the police nor the administration provide any possibility of solving major conflicts regarding the pastures because the grazing rights are not formalized.

Most of the pastoralists preferred to divide the winter pastures into customary tenures, but they disagreed on the use of voluntary agreements versus formalization through the courts. There were pastoralists who claimed that newly rich pastoralists do not respect the customary tenures, so these respondents only options was to use the court to claim pasture rights. Others argued that the voluntary division of pastures does not work due to overlapping use by different siida groups. Only 12% did not want to divide the winter pastures into smaller tenures related to siida groups. A few warned that such divisions would ruin the traditional culture, solidarity and flexible use of the pastures and move reindeer pastoralism toward ranching.

As much as 88% perceived the governance system as a necessary third party for formalized conflict resolution, monitoring and enforcement. The lack of impartial and fair treatment due to kinship or friendship among the co-management boards was the main source of distrust. Recent decisions associated with total allowable reindeer numbers, weighing programs, and the processes behind the division of winter pastures had also caused discontent with the administration. Most pastoralists argued for more impartiality, either by using co-management boards from neighboring regions or by transferring authority to the governor.

The pastoralists did not support increased autonomy for the summer siidas. The primary arguments against devolution were the need for regulating the competition between siidas on the winter pasture and the protection against encroachment. Those who partly agreed argued that self-determination could work under the condition that appropriate rules or customary tenures were established on the winter pastures. For some, the transfer of rulemaking authority to the summer siidas was undesirable, as the elites have too much power in the local boards, whereas others maintained that the reindeer are privately owned and they cannot tell their relatives to slaughter their herds. Many pastoralists perceived the 2007 Act that devolved the rulemaking authority to the siidas as a repudiation of liability, as the governing bodies caused the situation. The generous allocation of herding licenses and subsidies allowed too many pastoralists to accumulate herds on shared pastures during the 1980s. Combined with the definition of winter pastures as commons in the 1978 Act, which some pastoralists interpreted as open access, new herds and expansionism have resulted in crowding on the winter pastures.

## Discussion

Our results suggest that the match between the scale of self-organization and the scale of formal governance is a key condition for sustainable outcomes in a SES. The CBM was designed to delegate the rulemaking authority to the siidas, but the challenge remains in how the users self-organize at the higher nested levels, i.e. winter pastures. In our case, the collective action problems emerged on the large winter pastures shared by several competing siidas. Our findings were based on the combination of quantitative analyses of data from official databases and interview inquiries, linking structural variables with explanations of sustainable outcomes [3], [10], [15]. In the quantitative analyses, we found winter pasture size to be the key variable influencing sustainable outcomes (Table 1, Figure 2). The selection of the five largest winter pastures for the interview inquiries allowed us to analyze the explanatory mechanisms behind this result. The interviewed pastoralists agreed on the sustainability policies, but had a low degree of trust to other herders on the winter pastures and no conflict resolution mechanisms for pasture use among the siida groups. As a result, the Sámi pastoralists did not call for more autonomy at the lowest scale but instead requested customary tenures or an impartial authority to devise clear rules and sanctioning mechanisms. The multilevel co-management system has not filled this institutional gap [19], [42], [45], [46] and lacks sufficient trust to act as a neutral, objective and impartial authority.

The effect of pasture size on sustainability differed between seasonal pastures. Large winter pastures had a negative effect on the herders’ livelihood income, the proportion of calf slaughter and the slaughter weights of calves. No such relationships were evident on the summer pastures. Most summer pastures have well-defined boundaries, with usually one siida group having the exclusive rights to the pasture. Accordingly, the size of the summer pasture only had small effects on the sustainability indicators. These differences between seasonal pastures were also evident in the interview inquiry (Figure 3). On the five largest winter pastures, the low level of trust also corresponds with the negative reciprocity among herders as well as the higher costs of monitoring and herding reindeers on snowmobiles. Open conflicts are usually avoided as the outcome could be retribution, and for small herders, the best strategy is usually to move away from large herds to avoid losses of reindeer. The seasonal difference in the effect of pasture size, combined with the seasonal difference in trust (and cooperation) among herders, do support the view that a mis-match between the scale of self-organization and formal governance on the winter pasture is among the major reasons behind unsustainable outcomes in the SES. Unknown confounders, such as systematic differences in the biophysical conditions or herding practices, could have biased the quantitative analyses, and in particular the estimated effect of winter pasture size. However, the field study suggests that the effects of such biases were small. The reindeer herders do not ascribe to alternative herding practices or sustainability goals (Figure 3). They report low degree of trust and the weak institutional arrangements on the largest winter pastures as their major challenges (Figures 4 and 5).

Our assertion that collective action problems are crucial for understanding this pastoral SES, is also corroborated by the fact that a high stability in the social organization of herders and a high degree of equality among herders, in addition to small winter pasture size, had a positive effect on some of the sustainability indicators. Specifically, the quantitative analyses showed that pastoralists belonging to the same siida throughout the year slaughtered a higher proportion of calves compared to herders that changed the siida group seasonally. Calves are generally slaughtered before the herds move into the winter pastures, and calf slaughter will, as explained by the herders in the interviews, spare the winter pastures and reduce the risk of losses of weak animals under unfavorable winter conditions (see also [31], [32], [47]). On the other hand, calf slaughter will reduce the herd size and thereby the herders’ influence and ability to acquire pasture during winter. The indicator was therefore expected to be particularly sensitive to the degree of cooperation among herders. The equality with respect to herd size was important for the slaughter weight of calves, which indicate how well reindeer density is regulated by the siidas. Finally, the effect of winter pasture size was also evident for the economic benefits derived from the pastures. Controlled for herd size, small winter pastures was the only factor that contributed positively to income. The lower income on the largest winter pastures could be explained by strategies associated with non-market values [36], [38], [48], but in our interviews the pastoralists perceive production as a primary goal, which is necessary to cover the high expenses of reindeer herding [17], [19], [34].

As CBM is often based on the subsidiary principle, the nestedness of social organization is easy to ignore when policies are put into practice [2], [3], [5]. The mismatch of CBM with an appropriate scale has also been suggested as one of the reasons behind the persistent decline of common pastures in the drylands of Africa [23]. A multilevel co-management system regulates macro-scale mobility in Sámi pastoral ecosystems but has failed to regulate micro-scale use on the higher nested levels. The prescription has been more devolution to users, or “enforced self-organization,” as local boards now have full responsibility for crafting rules for the sustainable use of all of the seasonal pastures. As our results show, this reform toward self-determination is likely to work better when the winter pasture size is smaller. Large pastures require an impartial third party to regulate the competition between the siidas, as was also confirmed by another survey showing that only 1/5 of the pastoralists think they will be able to manage these pastures themselves, i.e., without external assistance [43]. The importance of this mismatch between governance and the siida organization is corroborated by the comparison with other regions in Norway, where pastoralists in general manage their smaller pastures more sustainably [19], [31], [46]. Similar collective action problems have also been observed in northernmost Finland, where herding cooperatives self-organize to manage pastures without impartial authorities to deal with conflicts and power inequalities [49].

Critics of CBM have also argued that the strong emphasis on formalized rules, rights and sanctioning mechanisms does not fit the flexible social organization needed in mobile pastoral systems [23]. High climatic variability and an unpredictable supply of resources demand flexible resource-sharing arrangements, which cannot be as easily delineated as secure tenure rights [17]–[19]. Although they acknowledge the need for flexibility under adverse weather conditions, most Sámi pastoralists prefer delineation of borders on the basis of customary rights to tenures [46]. There are, however, reciprocal norms for sharing pastures, particularly in the rim zones of the siida tenures. These norms typically depend on prior agreement [46], but the timing and duration of stay are also factors, along with the degree of trust between neighbors. The call for customary rights in the five largest winter pastures could be explained by the low levels of trusts. Trust is a key condition for negotiations and flexible use on winter pastures, which may have eroded as number of siidas and herds have increased.

CBM focus on the capabilities of pastoralist groups to establish their own rules, monitoring and sanctioning mechanisms for managing common pastures [4], [23]. The fit of CBM to Sámi pastoralist ecosystems depends on the scale of resource sharing, as well as the setting in which rulemaking authority has been transferred. Historically, the “big-push” economic policies rapidly modernized the Sámi pastoral ecosystems from a system of low living conditions and the use of simple technology to an SES of higher welfare, marked integration and the use of snowmobiles and ATVs (see Text S1, [19], [30]). The increase in number of pastoralists and herd expansion has subsequently resulted in pressures on the customary tenure systems, which have limited the adaptability of the SES [19], [20], [30], [46]. The challenges have been met by CBM policies that devolve rulemaking authority to the lowest level of organization. We found CBM to have a better fit to such a SES when pastoralists cooperate on smaller scales and when there are higher stability in siida partnership. The lack of trust and self-organization among siidas on larger scales often need higher level of governance for devising clear rules, conflict resolution and sanctioning mechanisms [1], [5], [7]. The co-management board did not fill this institutional gap which may explain the pastoralists request for well-defined boundaries, and clearer rules for pasture use devised by an impartial authority.

## Supporting Information

Text S1 **The text provides a more detailed description of community-based management of Sámi pastoral ecosystems in Norway.**
(DOCX)
