# Supplementary material for: Community-Based Management: Under What Conditions Do Sámi Pastoralists Manage Pastures Sustainably?
Source: PLoS One. 2012 Dec 11;7(12):e51187. doi: 10.1371/journal.pone.0051187 (PMC3519842; doi:10.1371/journal.pone.0051187)
Supplement: Text S1 — The text provides a more detailed description of community-based management of Sámi pastoral ecosystems in Norway. (DOCX) [file pone.0051187.s001.docx]

# Community-based management in the Sámi pastoral ecosystems

CBM focus on the capabilities of pastoralist groups to self-organize to manage common pastures by establishing clear rules and boundaries, monitoring and sanctioning mechanisms by users themselves [1,2]. The fit of CBM to Sámi pastoralist ecosystems depends on the scale of mobility and flexible use targeted by CBM, as well as the historical context in which rulemaking authority has been transferred [2]. The CBM policies in Norway have evolved through several steps. Before the mid 70’s the Norwegian government had limited ambitions to regulate the internal use and management of pastures by the Sámi pastoralists [3]. One exception is the formal delineation of summer pastures which was regulated pursuant to the 1933 Act. The early formalization of summer pastures could be explained by the expansion of competing interests on the coastal summer pasture areas, notably small-scale agriculture [4,5]. The 1978 Reindeer Husbandry Act established co-management boards to regulate pasture use, and number of reindeer and pastoralists [5-9]. The Act was revised in 1996 to strengthen the role and capacity of local boards to manage summer pastures, and to allow delineation of pastures into siida territories. However, the clearest reform towards CBM was the 2007 Reindeer Husbandry Act (Lov om reindrift 2007-06-15-40), which was developed by a law committee in which the majority were Sámi pastoralists.

The 2007 Act legally acknowledges the siida as the key unit of self-organization in the Sámi pastoral ecosystems [6,10,11]. The siida refers to a group of Sámi pastoralists, usually kinship groups, that collectively herd their reindeer together, both by seasonal migration of herds (i.e. macro-mobility) and by micro-mobility on winter pastures [8,13-16, 21]. The siida is defined by the: i) existence of a reindeer herd, ii) active herding and control of the herd by Sámi pastoralists and iii) that one or several families keep their reindeers in that cooperative [17]. It is a flexible institution as the number and composition of herds and herders change in relation to seasonal pastures and environmental variability [18,19, 21]. The flexibility to change siida partners is also argued by some authors to be more ecologically sustainable as the herd size could more easily be adapted to the variable pasture conditions and the labour available for managing the herds [17,18, 20, 21]. Such flexible relationship between pasture, herd size and labour is not only considered as ecologically sustainable, but is also argued to mitigate internal conflicts as households could easily leave the siida. It is also argued that flexibility must be balanced according to the need for stability, because of the site tenancy of reindeers, the need for knowledge about the pastures and the kinship groups attachment to the territory [17,18]. Historically, the siida system has also been associated with customary rights to resources, including the rights of winter siidas to core tenures with flexible, overlapping boundaries on winter pastures [8,16,17,24-26]. Albeit the claims of breakdown in these customary traditions caused by governmental regulations, such norms for allocation of pasture use still persists in the Sámi pastoral communities [25,26]. However, the stability of siida partners on seasonal pastures varies extensively in Finnmark. The summer siida is usually the largest unit of social organization and typically consists of several families, but whereas some siidas split up into smaller winter siidas, others have stable siida partnerships throughout the year.

The customary rules in the siida system regulate allocation of labour and pastures, but the slaughter and herd compositions have traditionally been decided upon by the individual reindeer families. Paine (1964) refers to this distinction as herding and husbandry [14]. The collective responsibility of siidas is associated with herding; the movement of reindeer between seasonal pastures, the gathering and separation of herds that have been inter-mixed, the active control and monitoring of herds on winter pastures as well as the maintenance of shared property such as fences and cabins. Husbandry, on the other hand, refers to the ownership, maintenance and management of reindeer by individual households, and involves activities such as slaughtering, earmarking and castration etc. [14,15,27]. Long-term decisions about herd size and composition, production, and household economy have usually not been in the reign of the siida, but the household, or the family head together with the spouse. Today this level of decision making is formalized by the 2007 Act as a siida unit, and the children as well as close relatives could own reindeer in the siida unit as identified by traditional earmarks. Siida and siida units with many family members will have a greater flexibility and access to labour in work intensive periods, than smaller families and siidas. A larger siida with many families need to balance the husbanders interests in increasing herd sizes and the solidarity among siida partners [6,8,17]. Unanimity and negotiations are preferred for making decisions in siidas, as well as for conflict mitigation with other siidas, but some have highly respected leaders, Siida isit, that influence decisions to a larger extent [4,5,15,19].

Flexible and resilient management of the pastoralist SES pastures is determined by both macro- and micro mobility [19,24]. In Scandinavia the macro-mobility of reindeer herding (*Rangifer t. tarandus L.*) has been strongly influenced by the closure of national borders [20,23]. The closure of the Finnish-Norwegian borders in 1852 resulted in loss of winter pastures for the Norwegian pastoralists, whereas the Finnish pastoralists lost the access to summer pastures and have moved more towards stationary free ranging systems, fencing and supplementary feeding [25]. In Sweden there are still seasonal movements between forest winter pastures and alpine summer pastures [23], whereas in Norway the transhumance pastoralism between inland winter pastures and coastal summer pastures still persists [26]. Today the seasonal movements in Norway are highly regulated by the co-management boards [27]. The reindeer families are assigned to specific summer and winter pastures for which the borders and grazing dates are decided upon by the national co-management board. Only Sámi pastoralists are entitled to reindeer herding, but the co-management boards regulate access by licensed siida units. The co-management boards have also been responsible for managing the number of pastoralists and the total allowable quota of reindeers on the pastures, but such regulations were not fully implemented before the revision of the Act in 2007 [7,29].

The rulemaking authority for managing summer and winter pastures (i.e. micro-mobility) has been transferred to local boards pursuant to the 2007 Act. Although the siida insitutions was not legally acknowledged until the 2007 Act, the CBM was initiated already in the mid-90’s by devolution to local boards for managing summer pastures [9,13,30]. In practice, the locally elected boards has had the responsibility to decide upon internal affairs on summer pastures, such as the allocation of pastures, reindeer, labour and other costs. A revision of the policies in mid-90’s aimed at formalizing the management of pastures by encouraging the development of formal plans by local boards. These policies were criticized for putting too much emphasis on devolution to the summer pastures, while ignoring the challenges on larger scales [31]. The boards have also been criticized for elite capture by large family clans that had more votes in the board [30,32]. The 2007 Act continued to craft CBM policies around the local boards on the summer pastures, and made these boards responsible for suggesting their own rules for the sustainable use of the pastures, which could be accepted or rejected by the co-management boards [29, 32]. Rejection of such prescriptive rules of usage by the Ministry of Agriculture and Food has been made on the basis of general sustainability principles, which include indicators such as slaughter weights, meat production per reindeer, and proportion of calves per female in the fall [3]. The sustainability principles have been developed by a task force consisting of managers, scientists and pastoralists [33]. To accomplish sustainable management of the pastoral SES, the siidas is supposed take into account pasture management on larger scales and the interconnectedness and the capacity of all seasonal pastures. Most local boards have now established rules, and fines and mandatory slaughter are among the sanction mechanisms for non-compliance in the 2007 Act.

As the CBM policies has mainly been focused on the local boards which manage the summer pastures, there is still a challenge to reach agreement about the use on winter pastures, which on the largest pastures is shared by several smaller winter siidas. Boards have been established for managing winter pastures, but so far only one of the boards have devised rules for pasture use in winter [3]. The summer pastures had well-defined borders existing long before the establishment of the co-management boards, but on winter pastures there were no external interests in the use of the land and the micro-mobility and pasture use in winter have mainly been regulated by the Sámi pastoralists themselves [4,5]. As a result the delineation of the largest winter pastures was only finalized by the national co-management board in 2004 [34]. There have, however, been early attempts by the managers to facilitate voluntary agreements within these winter pasture zones, and in some areas such agreements have existed since 1990 [35]. According to the report, the agreements have, however, not worked as intended due to unclear customary tenures, overlapping claims to pastures, and lack of enforcement mechanisms [35]. The reciprocal norms of pasture use is still alive in the Sámi pastoral communities [25,26], but as voluntary agreements did not bear fruits many pastoralists now turn to the courts to formalize their rights to siida tenures [25]. Mikkel Nils Sara, a research fellow at the Sami University College and a siida leader himself, argues that there has been too much emphasis on the sustainability criteria and the maximum allowable reindeer numbers (article 60 in the 2007 Act) and too little focus on the juridical approach to clarify siida rights to tenures on winter pastures (article 59) [23]. The latter solution, he argues, would to a larger extent ensure self-organization by siidas and allow Sámi to use their own traditional ecological knowledge to achieve sustainability. Such customary rights to core tenures could be set either through the Land Consolidation Court or a special commission [23,28,36].

One of the causes behind the weakened reciprocal norms on winter pastures is the rapid transitions from subsistence to monetary income and market integration of the Sámi pastoral communities [13,29]. “Big push” policies introduced in the mid-80’s to secure the welfare and income of Sámi pastoralists have rapidly resulted in increased herd sizes and recruitment to the livelihood [13,21,23,29]. The economic policies are regulated by negotiated agreements between the Government and the Saami Reindeer Herders’ Association, and include investments, such as in slaughter houses, cabins and fences, as well as direct subsidies to families Combined with a period of favourable winters, the reduced harvest resulted in an exponential increase in herds during the 80’s. At present the reindeer densities in the Finnmark are historically high [22], and at much higher levels than elsewhere in Scandinavia [12]. The customary tenures have also been put under pressure by the larger herds expanding into new areas, and the use of motorized vehicles for herding have allowed more extensive use of pastures [7,9,27]. This setting challenges the local boards ability to make their own rules and thus the implementation of CBM policies.

**References**

1. Ostrom E (1990) Governing the commons: The evolution of institutions for collective action. Cambridge: Cambridge University Press.

2. Turner MD (2011) The New Pastoral Development Paradigm: Engaging the Realities of Property Institutions and Livestock Mobility in Dryland Africa. Society & Natural Resources 24: 469-484.

3. Anonymous (2012) Riksrevisjonens undersøkelse av bærekraftig reindrift i Finnmark. Oslo: Stortinget

4. Bull KS, Oskal N, Sara MN (2001) Reindriften i Finnmark. Oslo: Cappelen akademisk.

5. Riseth JÅ (2003) Sami reindeer management in Norway. In: Jentoft S, Minde H, Nilsen R, editors. Resource management and global rights. Delft, Netherlands: Eburon Delft.

6. Ulvevadet B (2012) The governance of Sami reindeer husbandry in Norway. Tromsø: University of Tromsø.

7. Ulvevadet B, Hausner V (2010) Incentives and regulations to reconcile conservation and development: Thirty years governance of Sami pastoral ecosystems in Finnmark, Norway. Journal of Environmental Management 92: 2794-2802.

8. Kalstad JK (1999) Reindriftspolitikk og samisk kultur - en uløselig konflikt? Guovdageaidnu [Kautokeino]: Sámi instituhtta. 328 s. : ill. p.

9. Kalstad JKH (1998) Pastoralism and Management of Common Land in Sami Districts. In: Jentoft S, editor. Commons in a cold climate : coastal fisheries and reindeer pastoralism in North Norway : the co-management approach. Man and the biosphere series. New York: UNESCO ; Parthenon Pub.

10. Sara MN (2009) Siida and traditional sámi reindeer herding knowledge. Northern Review 30: 153-178.

11. Ravna Ø, Olli JJ (2011) Sedvanerettslige oppfatninger om arealbruk blant reindriftsutøvere. Guovdageaidnu [Kautokeino]: Sámi instituhtta. 72.

12. Pape R, Löeffler J (2012) Climate change, land use conflicts, predation and ecological degradation as chellenges for reindeer husbandry in Northern Europe: What do we really know after half a century of research? Ambio 41: 421-434.

13. Riseth JA, Vatn A (2009) Modernization and Pasture Degradation: A Comparative Study of Two Sami Reindeer Pasture Regions in Norway. Land Economics 85: 87-106.

14. Paine R (1964) Herding and Husbandry: two Basic Distinctions in the Analysis

of Reindeer Management Folk 6(1):83–88.

15. Paine R (1994) Herds of the Tundra. Washington: Smithsonian Institution Press.

16. Oskal N, Sara MN (2001) Reindriftssamiske sedvaner og rettsoppfatninger om land. Oslo: Statens forvaltningstjeneste.

17. Anonymous (2001) Forslag til endringer i reindriftsloven : innstilling fra Reindriftslovutvalget oppnevnt av Landbruksdepartementet 5. november 1998 : avgitt 15. mars 2001; Reindriftslovutvalget, Bull KS, Landbruksdepartementet, editors. Oslo: Statens forvaltningstjeneste

18. Sara MN, Knudsen A (2001) Reinen - et gode fra vinden. Karasjok: Davvi girji.

19. Oskal N (1997) Hva er reinlykke? Alta: Reindriftens fagråd.

20. Bjørklund I (1999) Den nasjonale integrasjon av det samiske reindriftssamfunn. Oslo: Ad notam Gyldendal.

21. Bjørklund I (1999) Sami reindeer herding : indigenous resource management in a changing world. S. 167-174 p.

22. Tømmervik H, Riseth JA (2011) Historiske tamreintall i Norge fra 1800-tallet og fram til i dag. NINA. 36 p.

23. Sara MN (2011) Land usage and siida autonomy. Arctic Review of Law and Politics 2011/2.

24. Hågvar G (2006) Den samiske rettsdannelse i indre Finnmark. Guovdageaidnu [Kautokeino]: Sámi instituhtta.

25. Ravna Ø, Olli JJ (2011) Sedvanerettslige oppfatninger om arealbruk blant reindriftsutøvere: Herunder oppfatninger om reintallsfastsetting og om behov for en rettsinstans som er egnet for å ordne beitebruk i reindriftsområder. Kautokeino: Sami University College.

26. Jonassen J, Kalstad AS (2003) Internrettslige betraktninger om reindriften : reindriften i skjæringspunktet mellom intern organisering og norsk rett. Røros: Advokatfirmaet Robertsen.

27. Pehrson RN (1964) The bilateral network of social relations in Könkämä Lapp District. Oslo: Norsk folkemuseum.

28. Ravna Ø (2004) Can land consolidation principles improve the land use situation in reindeer husbandry. S. 216-230 p.

29. Hausner V, Fauchald P, Tveraa T, Pedersen E, Jernsletten J-L, et al. (2011) The ghost of development past: the impact of economic security policies on Saami pastoral ecosystems. Ecology and Society 16.

30. Ulvevadet B (2008) Management of reindeer husbandry in Norway - power-sharing and participation Rangifer 28: 53-78.

31. Kalstad JKH (1998) Pastoralism and Management of Common Land in Sami Districts. In: Jentoft S, editor. Commons in a cold climate Coastal fisheries and reindeer pastoralism in North Norway: the co-management approach. New York: UNESCO, Parthenon Pub.

32. Kalstad JKH (1999) Reindriftspolitikk og samisk kultur- en uløselig konflikt? Et studie av reindriftstilpasninger of moderne reindriftspolitikk. Kautokeino: Nordic Sámi Institute.

33. Anonymous (2008) Kriterier/indikatorer på økologisk bærekraftig reindrift. Rapport fra arbeidsgruppe opprettet av Landbruks - og matdepartementet. Oslo: Landbruks - og matdepartementet.

34. Anonymous (2010) Ressursregnskap for reindriftsnæringen 08/09 (Ecological statistics of reindeer husbandry). Alta: Reindeer Husbandry Administration.

35. Olli JJ, Henriksen JB (2007) Prosjekt 514103. Sandvika: Asplan Viak.
